# Patient Contact Recall after SARS Exposure

**DOI:** 10.3201/eid1104.040648

**Published:** 2005-04

**Authors:** Popy Dimoulas, Karen A. Green, Altynay Shigayeva, Michael Aquino, Allison McGeer, Damon C. Scales, Toronto SARS Hospital Investigation Team

**Affiliations:** * Mount Sinai Hospital, University of Toronto, Toronto, Ontario

**Keywords:** dispatch, SARS, occupational exposure, intensive care unit, recall, reliability

## Abstract

We reinterviewed healthcare workers who had been exposed to a patient with severe acute respiratory syndrome (SARS) in an intensive care unit to evaluate the effect of time on recall reliability and willingness to report contact activities and infection control precautions. Healthcare workers reliably recalled events 6 months after exposure.

Severe acute respiratory syndrome (SARS) quickly spread within hospitals after it was first identified in Toronto, Canada, in March 2003. Healthcare workers who cared for severely ill patients with SARS were at high risk of acquiring an infection (*1*).

Risk factors associated with SARS transmission have been assessed by using retrospective data from medical chart reviews and healthcare worker interviews (*2**–**4*). Infection control practitioners routinely use this method to determine the degree of exposure to communicable diseases in hospitals, but its reliability and validity are unknown. To better understand the impact of time on recall reliability and healthcare workers' willingness to report infection control breaches, we reinterviewed a cohort of healthcare workers who had been exposed to a patient with SARS and who had previously been studied (*3*).

## The Study

During the first Toronto SARS outbreak in March 2003, 69 healthcare workers at risk for SARS were interviewed a median of 1.2 months (range 1 to 1.5 months) after exposure (*3*). Five months (range 4.8 to 5.3 months) after participating in this initial study, 30 of these healthcare workers were asked to participate in another study. These workers were eligible for participation in this second investigation because they had entered the index patient's room from 24 hours before intubation to 4 hours after intubation. Both investigations involved telephone or face-to-face interviews to determine the amount of time the worker had spent in contact with the patient, the activities that had occurred while the worker was in the patient's room, and the personal protective equipment used by the worker. The second questionnaire was more detailed than the first but contained a substantial number of questions that were identical to those in the first questionnaire.

Responses to identical questions in the initial and follow-up interviews were compared and expressed as proportions. Responses obtained during the initial interview were considered the reference standard for comparison with follow-up interview responses. Agreement between the initial and follow-up responses was quantified by using the kappa statistic and confidence intervals. The kappa statistic (κ) is a commonly used measurement of agreement or repeatability in epidemiologic studies. Kappa values from 0.20 to 0.39 indicated fair agreement, values from 0.40 to 0.59 indicated moderate agreement, values from 0.60 to 0.79 indicated good agreement, and values >0.80 indicated excellent agreement (*5*).

Twenty-seven of the 30 eligible healthcare workers agreed to the second interview (Table 1). The proportion of healthcare workers who reported the same exposure in the follow-up interview as during the initial interview was >80% for most respiratory and airway management activities and >90% for procedures such as vascular catheter insertion. However, the proportion of similar responses was lower for routine patient care activities such as bedding change (67%) and nebulizer treatments (70%) (Table 2).

**Table 1:** Characteristics of healthcare workers and severe acute respiratory syndrome (SARS) index patient contact

Characteristics*	No. (%) of healthcare workers (N = 27)
Demographic characteristics	
Age group (y)	
20–29	7 (26)
30–39	9 (33)
40–49	8 (30)
≥50	3 (11)
Sex: female	18 (67)
Occupation	
Nurse	10 (37)
Respiratory therapist	8 (30)
Other†	9 (33)
No. with laboratory-confirmed SARS	3 (11)
Contact characteristics	
Cumulative time spent in patient's room‡	
≤10 min	9 (35)
11–30 min	8 (31)
31 min–4 h	6 (23)
>4 h	3 (11)
Touched patient	16 (59)
Face ≤3 feet of patient	18 (67)
Contact with mucous membranes or respiratory secretions	7 (26)
Patient coughing or spitting while healthcare worker was present	11 (41)
Performed or assisted with intubation	2 (7)

*Characteristics ascertained from initial interview. †Other occupation categories include service assistants, residents, 1 physician, and 1 pharmacist. ‡Based on 26 healthcare worker responses.

**Table 2:** Reliability of healthcare worker recalling presence in a SARS patient's room during patient care activities*

Activity	No. (%) affirmative during initial interview	No. (%) same response on follow-up interview	No. (%) of responses overestimated	No. (%) of responses underestimated	No. (%) missing	Kappa	95% CI
Respiratory characteristics and airway management							
Intubation	2 (7)	25 (93)	1 (4)	1 (4)	0	0.46	0.00–1.00
Suctioning before intubation	2 (7)	24 (89)	2 (7)	1 (4)	0	0.34	0.23–0.91
Suctioning after intubation	2 (7)	25 (93)	2 (7)	0	0	0.63	0.17–1.00
Manipulation of oxygen face mask/tubing	13 (48)	22 (82)	3 (11)	2 (7)	0	0.70	0.44–0.97
Manual ventilation	2 (7)	25 (93)	2 (7)	0	0	0.63	0.17–1.00
Mechanical ventilation	6 (22)	24 (89)	2 (7)	1 (4)	0	0.70	0.38–1.00
Patient coughing/spitting while healthcare worker present	11 (41)	16 (59)	8 (30)	0	3 (11)	0.38	0.11–0.66
Routine patient care							
Changing bedding	11 (41)	18 (67)	2 (7)	7 (26)	0	0.26	0.09–0.60
Bathing patient	4 (15)	26 (96)	1 (4)	0	0	0.87	0.61–1.00
Emptying urinary catheter collection bag or collecting urine sample	4 (15)	25 (93)	0	2 (7)	0	0.63	0.17–1.00
Oral care or nasal swab	4 (15)	25 (93)	1 (4)	1 (4)	0	0.71	0.32–1.00
Stool sample or rectal swab	0	25 (93)	1 (4)	0	1 (4)	–	–
Nebulizer treatments	0	19 (70)	6 (22)	0	2 (7)	–	–
Procedures							
BiPAP	19 (70)	18 (67)	2 (7)	6 (22)	1 (4)	0.32	0.01–0.66
Electrocardiogram	3 (11)	23 (85)	3 (11)	1 (4)	0	0.42	0.04–0.88
Insertion of central venous catheter	0	27 (100)	0	0 (0)	0	–	–
Insertion of peripheral intravenous catheter or arterial catheter	5 (18)	25 (93)	1 (4)	1 (4)	0	0.75	0.43–1.00
Insertion of nasogastric tube	0	26 (96)	1 (4)	0	0	–	–
Insertion of urinary (Foley) catheter	0	27 (100)	0	0	0	–	–
Chest physiotherapy	0	26 (96)	1 (4)	0	0	–	–
Other							
Cleaning room/furniture	2 (7)	26 (96)	0	1 (4)	0	0.65	0.02–1.00
Cleaning medical equipment	3 (11)	22 (82)	2 (7)	1 (4)	2 (7)	0.51	0.03–0.99

*SARS, severe acute respiratory syndrome; CI, confidence interval; BiPAP, bi-level positive air pressure.

Agreement between initial and follow-up responses was high for most respiratory and airway management activities, including suctioning after intubation (κ = 0.63), manipulation of oxygen face mask or tubing (κ = 0.70), manual ventilation (κ = 0.63), and mechanical ventilation (κ = 0.70). Agreement was fair to moderate for the following respiratory procedures: intubation (κ = 0.46), suctioning before intubation (κ = 0.34), and patient coughing while the healthcare worker was in the room (κ = 0.38). Agreement was high for routine patient care activities, including emptying urinary catheter collection bag or collecting urine sample (κ = 0.63), bathing the patient (κ = 0.87), and performing oral care or obtaining nasal swabs (κ = 0.71). Agreement was also high for inserting an arterial line (κ = 0.75) and for cleaning the patient's room (κ = 0.65).

Healthcare workers were asked during both interviews to estimate whether they had spent >10 minutes, >30 minutes, or >4 hours in the patient's room. Twenty-two (88%) of the 25 healthcare workers that participated in both interviews provided the same estimates of exposure duration. Two healthcare workers overestimated and 1 underestimated the time spent in the patient's room. Kappa values (κ = 0.52) did not vary according to the duration of exposure.

Relative to their initial responses, on follow-up, healthcare workers tended to overestimate their presence in the patient's room during respiratory and airway management activities, particularly nebulization therapy. However, during the second interview, they were less likely to report being in the room while a bi-level positive air pressure unit was being used or while bedding was being changed. The rates of overestimated responses versus underestimated responses for other patient care activities were similar (Table 2). Healthcare workers who subsequently developed cases of laboratory–confirmed SARS were not more or less likely to remember their presence or absence during patient care activities (data not shown).

In the hospital, use of additional precautions (gown, gloves, and surgical masks for room entry) for methicillin-resistant *Staphylococcus aureus* was practiced by the healthcare workers (*6*). Compliance varied among healthcare workers, but the proportion of workers with the same response during the follow-up interview was >80% for all infection control precautions, except wearing a gown (76%, data not shown). In general, responses in the 2 interviews showed little variation in infection control precautions.

## Conclusions

Our results indicate that healthcare workers in this study reliably recalled contact practices, patient care activities, and infection control precautions 5 months after their initial interview and 6 months after exposure to a patient with SARS. The proportion of identical follow-up responses averaged >85% for contact practices, patient care activities, and infection control precautions. Agreement between initial and follow-up responses was good to excellent for most respiratory practices and airway management activities, routine patient care activities, and other medical procedures.

The lowest proportion of identical responses observed on the initial and follow-up interview was for being in the patient's room while the patient was coughing or spitting (59%), with a kappa value (0.38) indicating fair agreement. The risk of droplet and airborne spread of communicable diseases is assumed to be greater if a patient is frequently coughing. Hence, different infection control precautions have been recommended when caring for patients who are coughing (*7*). However, our results suggest that recollection of contact during this activity may not be reliable. Whether this poor reliability is related to the effect of time on memory or the intermittent nature of coughing is unclear.

The inferences that can be drawn from this study are limited by the relatively small size of our cohort. Caring for patients with SARS can be a memorable and frightening event (*8**,**9*), and recall reliability in our study may not be generalized to other clinical situations. Furthermore, the similarities among questions during the 2 interviews may have resulted in the potential for recall bias, causing an overestimation of reliability within respondents (*10*). Finally, our study measured the reliability rather than the validity of healthcare worker recall for determining exposure risk. Nonetheless, our findings that healthcare workers reliably recalled exposure after several months following the event should be reassuring to investigators studying risk factors for SARS transmission in hospitals and to infection control practitioners assessing exposure to communicable diseases.
